# Biomarkers of Alcohol Use in Pregnancy

**Published:** 2004

**Authors:** Cynthia F. Bearer, Joan M. Stoler, Janine D. Cook, Siri J. Carpenter

**Affiliations:** Cynthia F. Bearer, M.D., Ph.D., is an associate professor in the Departments of Pediatrics and Neurosciences at Case Western Reserve University, Rainbow Babies and Children’s Hospital, University Hospitals of Cleveland, Cleveland, Ohio. Joan M. Stoler, M.D., is an assistant professor of pediatrics, Harvard Medical School and Massachusetts General Hospital, Boston, Massachusetts. Janine D. Cook, Ph.D., is an assistant professor in the Department of Medical and Research Technology, School of Medicine, University of Maryland, Baltimore, Maryland. Siri J. Carpenter, Ph.D., is a science editor for *Alcohol Research & Health*

Alcohol use during pregnancy is a significant public health problem. Although most women who drink before pregnancy substantially reduce their consumption or completely stop drinking once they become pregnant, approximately 14 to 22.5 percent of women report drinking some alcohol during pregnancy (Bearer 2001).

The costs of prenatal alcohol use are high. Risky drinking (defined as more than seven standard drinks^1^ per week or five or more standard drinks on a drinking day [CDC 2002]) during pregnancy is a primary risk factor for fetal alcohol syndrome (FAS), the most common preventable cause of mental retardation.^2^ Prenatal alcohol exposure also can result in fetal alcohol spectrum disorders (FASD), in which the affected children do not show the classical FAS pattern but nonetheless exhibit mental, developmental, behavioral, and social deficits as well as other birth defects. Some evidence indicates that even low-risk drinking (defined as fewer than seven standard drinks per week or three or fewer standard drinks per drinking day [NIAAA 1995]) during pregnancy can cause adverse fetal effects, but how this damage occurs is not fully understood (Bearer 2001). An estimated 1 percent of all live-born infants show some prenatal alcohol-related damage, contributing to societal costs estimated at between $75 million and $9.7 billion per year (May and Gossage 2001).

Because of alcohol’s adverse effects on the fetus, all women should be counseled to refrain from drinking during pregnancy. Tests that could identify women who continue to drink while pregnant—and could detect the effects of alcohol exposure on the developing fetus or newborn—would be invaluable for several reasons. Identifying these women would facilitate interventions that could help them stop using alcohol during pregnancy, thereby minimizing alcohol’s effects on fetal brain development. Even discovering prenatal alcohol use later in pregnancy or soon after birth would be important because it would identify infants who are at risk for alcohol-associated birth defects and could make it possible to monitor them for potential problems, facilitate a more stable living environment, and provide special services if needed (Bearer et al. 1999; Stoler and Holmes 1999, 2004). In particular, identifying at-risk children before age 6 reduces the likelihood of secondary problems associated with FAS and FASD, such as mental health problems, school failure, delinquency, inappropriate sexual behavior, and alcohol and other drug problems (Streissguth et al. 1996). In addition, interventions that help new mothers reduce problem drinking could enhance their ability to care for their children and reduce the risk of alcohol problems during their subsequent pregnancies (Russell et al. 1996).

Developing effective biomarkers of prenatal alcohol use also may promote better scientific understanding of alcohol effects that occur with different patterns of maternal alcohol use during pregnancy.

## Maternal Self-Report

Currently no laboratory test can identify and quantify prenatal alcohol use that takes place over a protracted period. Because alcohol itself and the main product of its metabolism, acetaldehyde, break down rapidly in the blood, they cannot be used to distinguish between a single drinking episode and chronic, intermittent alcohol use. Testing blood, breath, or urine is useful only for assessing very recent alcohol exposure. Because biological markers currently in use may not be effective in screening for risky alcohol use occurring over the longer term, such as during pregnancy, clinicians most commonly use brief screening measures that rely on maternal self-reports to assess drinking patterns (Chang 2001; Russell et al. 1994, 1996; Savage et al. 2002).

Major disadvantages of such screening measures are that it often is difficult for people to recall the amount and frequency of their alcohol intake, and the stigma and fear of punishment (e.g., incarceration or involuntary commitment) associated with drinking alcohol during pregnancy can make women reluctant to reveal prenatal alcohol use, especially if they drink heavily (Bearer et al. 2003; Chan et al. 2003).

Some screening instruments attempt to circumvent pregnant women’s reluctance to disclose prenatal alcohol use by including questions that assess prenatal alcohol use indirectly—for example, by asking women to report the number of drinks they can consume before passing out or falling asleep (Russell et al. 1994, 1996). Some research indicates that such screening instruments can effectively flag heavy drinking. However, they do not provide a long-term, objective measure of the full range of prenatal alcohol use. Supplementing these measures with a biological marker for prenatal alcohol use would allow earlier identification and intervention for exposed infants and would make it easier to recognize women who are at risk for drinking during their next pregnancy (Bearer et al. 2001, 2003).

## Biological Samples for Detecting Drinking During Pregnancy

Traditionally, samples of neonatal or maternal urine and blood have been used to determine prenatal alcohol use (Halmesmäki et al. 1992; Wisniewski et al. 1983). However, the biomarkers that have been measured in these samples mostly reflect alcohol exposure only in the 2 to 3 days before delivery. Neonatal urine is difficult to collect, and blood collection for a neonate is an invasive procedure. Other samples that could be used after delivery to assess biomarkers of prenatal alcohol consumption include amniotic fluid, cord blood, neonatal hair, placenta, breast milk, the infant’s first fecal material (meconium), and the cheese-like material that covers the skin of a fetus (vernix). (The table summarizes information on potential maternal, fetal, and newborn samples.)

The usefulness of different fluid and tissue samples depends not only on their biochemical suitability but also on how readily they can be collected and how large the resulting sample is (Bearer 2001). For example, although amniotic fluid, cord blood, and placenta afford large samples, the window of opportunity for obtaining them is narrow. Likewise, analyses of maternal or neonatal hair can indicate the timing of prenatal alcohol use, but samples may not be available, either because a parent does not consent or, in the case of neonatal hair, because the newborn has none to give.

Meconium, most frequently used to detect prenatal exposure to drugs such as cocaine, holds particular promise for assessing prenatal alcohol use. Several studies have used meconium to measure concentrations of fatty acid ethyl esters (FAEEs), one promising biomarker of prenatal alcohol use (Bearer et al. 1999, 2003; Chan et al. 2003; Niemela et al. 1991). (See “Fatty Acid Ethyl Esters,” below.) Meconium is available beginning shortly after birth, and large samples can be collected directly from the infant’s diaper. Moreover, meconium begins to form as early as the 13th week of pregnancy and accumulates thereafter. Thus, using meconium sampled after delivery, it may be possible to obtain a detailed history of prenatal alcohol use over a longer period than is the case for biological samples such as neonatal urine or cord blood (Bearer et al. 1999, 2003; Chan et al. 2003).

A disadvantage of meconium as a matrix for detecting biomarkers of prenatal alcohol use is that it is present only for the first 2 or 3 days after birth. In contrast, neonatal hair, if present, can reflect maternal prenatal alcohol use for up to 2 or 3 months after birth; and the mother’s hair may be obtained throughout pregnancy to determine prenatal alcohol use during the pregnancy.

## Promising Biomarkers

Although some biomarkers may directly detect the effect of alcohol on the fetus or newborn (e.g., Bearer et al. 2000, 2001; Stoler et al. 1998), more progress has been made in developing biomarkers for maternal alcohol use during pregnancy. Research examining such biomarkers typically assesses their ability to correctly identify pregnant women who use alcohol (sensitivity) as well as pregnant women who do not (specificity).

**Table:** Biological Samples in Which Biomarkers Indicating Prenatal Exposure Could Be Measured

Sample	Advantages	Disadvantages*
**Maternal samples**		
Urine	Large sample size	Tampering possible
Hair	May indicate timing of exposure	May not be desirable, requires special analytical techniques
Blood	Battery of biomarkers may be used	Invasive, painful
Breath	Easy to obtain large quantities	Requires special equipment, technology is limited
Saliva	Easy to obtain	Sample size limited
Gases acquired through skin patch	Easy to obtain	Requires special equipment, technology is limited
Breast milk	Large sample, noninvasive	Narrow window of opportunity to collect if colostrum (or “first milk”) is required
**Fetal samples**		
Blood	Measures proximal exposure	Sample size limited, extremely invasive
Chorionic villus	Measures very early exposure	Sample size limited, extremely invasive
Amniotic fluid	Can be sampled from 18 weeks on	Sample size limited, extremely invasive
Newborn**		
Cord blood	Large volume available, discarded sample, battery of biomarkers may be used	Narrow window of opportunity to collect, single time point for measurement
Placenta	Large sample size, discarded sample	Narrow window of opportunity to collect
Umbilical cord	Large sample size, discarded sample	Narrow window of opportunity to collect
Amniotic fluid	Large sample size, discarded sample	Difficult to collect, narrow window of opportunity to collect
Urine	Concentrates metabolites, discarded sample	Difficult to collect
Hair	May indicate timing of exposure	May not be available, may not be acceptable to parent
Breath	Easy to obtain	Requires special equipment, technology is limited
Saliva	Easy to obtain	Small sample
Vernix	Discarded sample	Narrow window of opportunity to collect, not present on all babies, little background information available
Gases acquired through skin patch	Easy to obtain	Requires special equipment, technology is limited
Meconium	Easy to obtain, discarded sample, may indicate timing of exposure	Does not measure exposure prior to 2nd trimester

* All screening methods require consent.

** Obviously, biomarkers measured in newborn samples only indicate prenatal exposure retrospectively.

### Blood/Urine Marker Batteries

In an effort to develop a definitive biomarker for prenatal alcohol use, investigators have explored several biochemical changes associated with alcohol use, which can be detected by blood and/or urine tests:

- Long-term alcohol use causes the liver to release an enzyme called gamma-glutamyltransferase (GGT) into the bloodstream.
- Blood concentrations of variants of the protein transferrin (carbohydrate-deficient transferrin, or CDT) increase after alcohol consumption.
- An increase in the average size of red blood cells (i.e., mean corpuscular volume, or MCV) can signal excessive alcohol consumption.
- High levels of chemicals known as dolichols are found in the urine of alcoholics and are measurable in the urine of newborns whose mothers drank heavily during pregnancy (Halmesmäki et al. 1992; Wisniewski et al. 1983).
- When alcohol breaks down, its major metabolite, acetaldehyde, binds with hemoglobin in the blood, forming compounds known as hemoglobin–acetaldehyde adducts. These compounds remain in the blood longer than free acetaldehyde, making them a potential marker for long-term alcohol use (Niemelä et al. 1991).

Although these biological changes are useful indicators of risky drinking, no single marker is diagnostically sensitive and specific enough to be considered a definitive biomarker for prenatal alcohol use (Bearer 2001; Stoler et al. 1998). Several investigations have used panels of two or more blood markers in an effort to achieve greater sensitivity and specificity (Halmesmäki et al. 1992; Sarkola et al. 2000; Stoler et al. 1998). For example, in a study of 529 women, Stoler and colleagues (1998) found that a combination of CDT, MCV, GGT, and hemoglobin–acetaldehyde adducts was more strongly correlated with prenatal alcohol use (assessed by evaluating newborns for facial features indicating the effects of prenatal alcohol exposure) than was any single biomarker or the maternal self-report of alcohol use during pregnancy.

Another study of pregnant women who drank eight or more drinks per week found that hemoglobin–acetaldehyde adducts and CDT were not associated with the reported level of drinking (Sarkola et al. 2000). However, MCV and GGT were significantly higher in women drinking at least eight drinks per week compared with those drinking fewer than eight drinks per week. The specificity and sensitivity of these markers were not reported. Other studies have similarly suggested that using multiple biomarkers for prenatal alcohol use may be effective, but the sensitivity and specificity of combined blood markers, and thus their clinical usefulness, remain unclear.

**Figure:**
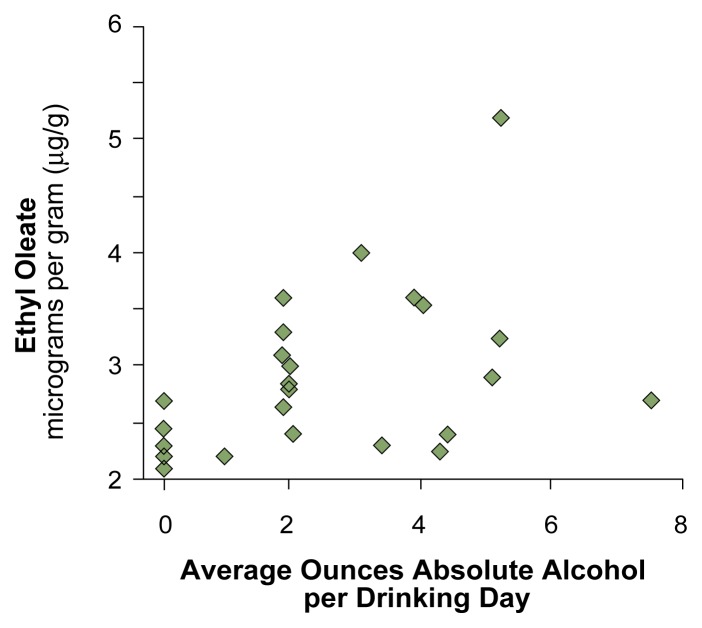
Relation of drinks per occasion during pregnancy to the amount of ethyl oleate concentration found in meconium (on a dry-weight basis). One ounce equals two standard drinks. Each diamond in the figure represents a mother participating in the study. Note that there are fewer diamonds in the upper left corner (indicating high specificity) and lower right corner (indicating high sensitivity). SOURCE: Bearer et al. 2003.

### Fatty Acid Ethyl Esters (FAEEs)

Fatty acid ethyl esters are metabolic products that result from the interaction between alcohol and fatty acids. FAEEs have been detected in the cord blood, meconium, and hair of infants; in other organs in adults; and in the placenta in mice (Bearer et al. 1999). Compared with alcohol and acetaldehyde, FAEEs have a prolonged half-life and show promise as biomarkers for prenatal alcohol use (Bearer et al. 1999, 2003; Chan et al. 2003; Klein et al. 1999). In a recent study, the presence of one FAEE (ethyl oleate) in meconium was strongly associated with self-reported drinking during the second and third trimesters of pregnancy, with very high sensitivity and specificity (see the figure) (Bearer et al. 2003). Further, the study found that FAEE concentration in meconium was more strongly related to the mother’s self-reported alcohol consumption per occasion than to the overall average she consumed per week. This finding may prove especially useful, as recent research has suggested that the number of drinks per occasion may be the best indicator of risk for alcohol-related impairment (CDC 2002).

Recently, a study found elevated FAEE concentrations in the hair of a newborn whose mother was alcoholic, as well as in the mother’s hair (Klein et al. 2002). Although preliminary, this finding indicates that both maternal and neonatal hair may be a useful matrix for examining FAEE concentrations.

The correlation between FAEEs in meconium and prenatal alcohol use is not perfect (Bearer et al. 2003; Chan et al. 2003). There probably are several reasons for this: (1) FAEEs may accumulate unevenly in meconium over time, so that samples do not appear to reflect reported drinking; (2) genetically determined variations in alcohol metabolism may influence the synthesis of FAEEs; and (3) illness or the use of some medications and food additives may affect FAEE concentrations. In order to set appropriate cutoff concentrations of FAEEs for analyses, a better understanding of these issues is needed.

## Proteomics

A number of proteins are known to be affected, either directly or indirectly, by alcohol. The rapidly advancing field of proteomics^3^ offers promise for developing sophisticated biomarkers that can detect very subtle biological changes associated specifically with alcohol use (Anni and Israel 2002), or can distinguish between currently drinking women who will continue to drink during pregnancy and those who will stop (Neuhold et al. 2004).

So far, few investigators have focused attention on proteomic analyses designed to establish potential biomarkers for prenatal alcohol exposure. Robinson and colleagues (1995), in a preliminary study, searched for serum protein variations associated with FAS. These researchers studied 12 children diagnosed with FAS (indicating that prenatal alcohol exposure had to have occurred) and 8 control subjects. Their analysis found eight proteins whose concentrations differed significantly between the two groups of children. No single protein distinguished children with FAS from children in the control group, but analyses revealed clusters of proteins that collectively distinguished between the two groups. This study demonstrates the power of looking at patterns of response to alcohol, and the approach it describes can be used as a strategy not only to identify children with prenatal alcohol damage but also women who, because of their biological response to drinking, may be at risk for drinking during their pregnancies.

Robinson and colleagues’ results, although not conclusive, illustrate the exciting potential of proteomic analysis to uncover novel biomarkers of both alcohol use and risk for drinking during pregnancy. The development of such proteomic biomarkers would be a major step toward primary prevention of the disorders associated with prenatal alcohol use.

## Conclusion

Currently, no laboratory test can definitively detect and quantify prenatal alcohol use. Developing effective biomarkers is an important step toward identifying at-risk pregnancies, preventing alcohol-related birth defects, and diagnosing and intervening with infants who may be at risk for later problems because of prenatal alcohol exposure. Several lines of research are promising, but much research is still needed to validate potentially useful biomarkers and identify further areas of possibility.

## Footnotes

1 The National Institute on Alcohol Abuse and Alcoholism (NIAAA) defines a standard drink as 11–14 g of alcohol, which corresponds to approximately one 1.5-oz shot of 80-proof alcohol (about 14 g alcohol), one 5-oz glass of table wine (11 g), or one 12-oz beer or wine cooler (12.8 g).

2 FAS is identified by its characteristic facial dysmorphology, growth restriction, and central nervous system/neurodevelopmental abnormalities (Sokol et al. 2003).

3 Proteomics is the study of how populations of proteins are coded and synthesized in the body, their interactions, their quantification, and their alteration in the presence of illness or other pathology.
